# Teaching Family Nurse Practitioner Students to Provide Mental Health Care to Older Adults

**DOI:** 10.1093/geroni/igaa057.034

**Published:** 2020-12-16

**Authors:** Victoria Grando, Roy Grando

**Affiliations:** Saint Luke's College of Health Sciences, Columbia, Missouri, United States

## Abstract

In recent years, FNPs have been challenged to deliver mental health services in the primary care setting. Over half of mental health services are provided in primary care, and one-quarter of all primary care patients have a mental disorder. Moreover, 20% of older adults have a mental or neurological disorder often not diagnosed. Nationally, it is estimated that 17% of older adults commit suicide, 15% have a mental condition, 11% have dementia, and 5% have a serious mental condition. There is a paucity of adequately prepared primary care providers trained in geropsychiatric treatment. A didactic course was developed to instruct FNP students in the skills needed to provide mental health treatment in primary care. We discuss mental illness in the context of culture to ensure that treatment is congruent with a patient’s unique cultural background and experiences. This shapes the patients’ beliefs and behaviors that influence the way they view their condition and what they perceive as acceptable solutions. We then go into detail about the common mental conditions that older adults exhibit. Through the case study method, students learn to identify the presenting problem, protocols for analyzing the case, which includes making differential diagnoses and a treatment plan including initial medications, non-medical treatments, and referral. Students are introduced to the DMS-5 to learn the criteria for mental health diagnosis with an emphasis on suicide, depressive disorders, anxiety disorders, bipolar disorders, substance use disorders, and neurocognitive disorders. We have found that students most often misdiagnose neurocognitive disorders.

